# AGRICOH: A Consortium of Agricultural Cohorts

**DOI:** 10.3390/ijerph8051341

**Published:** 2011-04-29

**Authors:** Maria E. Leon, Laura E. Beane Freeman, Jeroen Douwes, Jane A. Hoppin, Hans Kromhout, Pierre Lebailly, Karl-Christian Nordby, Marc Schenker, Joachim Schüz, Stephen C. Waring, Michael C.R. Alavanja, Isabella Annesi-Maesano, Isabelle Baldi, Mohamed Aqiel Dalvie, Giles Ferro, Béatrice Fervers, Hilde Langseth, Leslie London, Charles F. Lynch, John McLaughlin, James A. Merchant, Punam Pahwa, Torben Sigsgaard, Leslie Stayner, Catharina Wesseling, Keun-Young Yoo, Shelia H. Zahm, Kurt Straif, Aaron Blair

**Affiliations:** 1 Section of Environment and Radiation, International Agency for Research on Cancer, 150 Cours Albert Thomas, 69372 Lyon CEDEX 08, France; E-Mails: schuzj@iarc.fr (J.S.); ferro@iarc.fr (G.F.); straif@iarc.fr (K.S.); 2 Section of IARC Monographs (KS), International Agency for Research on Cancer, 150 Cours Albert Thomas, 69372 Lyon CEDEX 08, France; 3 Division of Cancer Epidemiology and Genetics, National Cancer Institute, 6116 Executive Blvd 7004, Rockville, MD 20852, USA; E-Mails: freemala@mail.nih.gov (L.E.B.F.); alavanjm@mail.nih.gov (M.C.R.A.); zahms@exchange.nih.gov (S.H.Z.); blaira@exchange.nih.gov (A.B.); 4 Centre for Public Health Research, School of Public Health, Massey University, P.O. Box 756, Wellington 6140, New Zealand; E-Mail: j.douwes@massey.ac.nz; 5 Epidemiology Branch, National Institute of Environmental Health Sciences, Research Triangle Park, 12 Davis Dr., Durham, NC 27703, USA; E-Mail: hoppin1@niehs.nih.gov; 6 Environmental Epidemiology Division (IRAS), Utrecht University, P.O. Box 80125, 3508 TC Utrecht, The Netherlands; E-Mail: h.kromhout@uu.nl; 7 Centre Régional de Lutte Contre le Cancer François Baclesse, Université de Caen, Esplanade de la Paix, 14000 Caen, France; E-Mail: p.lebailly@baclesse.fr; 8 National Institute of Occupational Health, P.O. Box 8149 Dep, N-0033 Oslo, Norway; E-mail: karl.c.nordby@stami.no; 9 Department of Public Health Sciences, School of Medicine, University of California, Davis One Shields Avenue Davis, CA 95616, USA; E-Mail: mbschenker@ucdavis.edu; 10 Marshfield Clinic Research Foundation, Epidemiology Research Centre, Webmaster, RL4, 1000 N. Oak Ave., Marshfield, WI 54449, USA; E-Mail: waring.stephen@mcrf.mfldclin.edu; 11 Epidemiology of Allergic and Respiratory Diseases Department, UMR S 707 INSERM & UPMC Paris 6, Medical School Saint-Antoine, 27, rue Chaligny, 75571 Paris CEDEX 12, France; E-Mail: annesimaesano@gmail.com; 12 Institut de Santé Travail Environnement, Université Bordeaux, 351 cours de la Libération, 33405 TALENCE Cedex, Bordeaux, France; E-Mail: Isabelle.Baldi@isped.u-bordeaux2.fr; 13 Centre for Occupational and Environmental Health Research, School of Public Health and Family Medicine, University of Cape Town, Private Bag X3, Rondebosch 7701, Cape Town, South Africa; E-Mails: Aqiel.Dalvie@uct.ac.za (M.A.D.); Leslie.London@uct.ac.za (L.L.); 14 Unité Cancer et Environnement, Centre Léon Bérard, 28 rue Laennec, 69008 Lyon, France; E-Mail: fervers@lyon.fnclcc.fr; 15 Cancer Registry of Norway, Institute of Population-Based Cancer Research, P.O. Box 5313 Majorstuen, N-0304 Oslo, Norway; E-Mail: Hilde.Langseth@kreftregisteret.no; 16 Department of Epidemiology, The University of Iowa, 2222 Old Hwy 218 S, Iowa City, IA 52242, USA; E-Mail: charles-lynch@uiowa.edu; 17 Cancer Care Ontario, 620 University Avenue Toronto Ontario M5G 2L7, Canada; E-Mail: John.Mclaughlin@cancercare.on.ca; 18 College of Public Health, General Hospital, The University of Iowa, 2222 Old Hwy 218 S, Iowa City, IA 52242, USA; E-Mail: james-merchant@uiowa.edu; 19 Department of Community Health and Epidemiology, Institution of Agricultural Rural and Environmental Health, University of Saskatchewan, Royal University Hospital, 300, 410-22nd Street East, Saskatoon SK S7K 5T6, Canada; E-Mail: pup165@mail.usask.ca; 20 School of Public Health, Department of Environmental & Occupational Medicine, University of Aarhus, Nordre Ringgade 1, DK-8000 Aarhus C, Denmark; E-Mail: Sigsgaard@dadlnet.dk; 21 Division of Epidemiology and Biostatistics, School of Public Health, University of Illinois at Chicago, 1603 West Taylor Street, Chicago, IL 60612, USA; E-Mail: lstayner@uic.edu; 22 Central American Institute for Studies on Toxic Substances (IRET), Universidad Nacional, Heredia, Calle 9, Avenidas 0 y 9 86-3000, Costa Rica; E-Mail: inekewesseling@gmail.com; 23 Department of Preventive Medicine, Seoul National University College of Medicine, 103 Daehangno, Chongno-gu, Seoul 110-799, Korea; E-Mail: kyyoo@plaza.snu.ac.kr

**Keywords:** agriculture, cohort studies, consortium, pesticides, occupational exposures

## Abstract

AGRICOH is a recently formed consortium of agricultural cohort studies involving 22 cohorts from nine countries in five continents: South Africa (1), Canada (3), Costa Rica (2), USA (6), Republic of Korea (1), New Zealand (2), Denmark (1), France (3) and Norway (3). The aim of AGRICOH, initiated by the US National Cancer Institute (NCI) and coordinated by the International Agency for Research on Cancer (IARC), is to promote and sustain collaboration and pooling of data to investigate the association between a wide range of agricultural exposures and a wide range of health outcomes, with a particular focus on associations that cannot easily be addressed in individual studies because of rare exposures (e.g., use of infrequently applied chemicals) or relatively rare outcomes (e.g., certain types of cancer, neurologic and auto-immune diseases). To facilitate future projects the need for data harmonization of selected variables is required and is underway. Altogether, AGRICOH provides excellent opportunities for studying cancer, respiratory, neurologic, and auto-immune diseases as well as reproductive and allergic disorders, injuries and overall mortality in association with a wide array of exposures, prominent among these the application of pesticides.

## 1. Rationale

Agricultural worker populations in many countries show distinctive exposure and disease profiles. These populations appear to have lower risk of some diseases such as colon and lung cancer, cardiovascular disease and allergic disease, which has been attributed to frequent exposure to microbial agents and healthier habits, including reduced tobacco use and increased physical activity [1–5]. On the other hand, regular exposure to certain pesticides, UV radiation, diesel exhaust and solvents and high dust levels has been reported to be associated with increased morbidity, including risk of several cancer types [6], respiratory disease including non-allergic asthma and chronic obstructive pulmonary disease (COPD) [7,8], neurotoxic [9] and reproductive outcomes [10]. Studies of agricultural populations are of great interest in their own right (*i.e.*, agricultural workers make up a large proportion of the working population worldwide), but they also contribute to a better understanding of disease risks associated with pesticides, other chemical, biological, and physical hazards for the general population because those exposures also occur outside agriculture. Additionally, these studies are suited to identify factors that may protect against particular types of cancer as well as allergies and other non-malignant conditions. Indeed, studies of agricultural populations have the potential to inform effective interventions to reduce disease burden in the general population.

## 2. Background

In 2006, the US-National Cancer Institute (US-NCI) brought together principal investigators of a number of agricultural cohorts to develop an international agricultural cohort consortium to study cancer and other health outcomes in association with agricultural exposures. Investigators from thirteen cohorts from Canada, France, New Zealand, Norway, Republic of Korea and USA attended this first workshop. To foster a transition into an active consortium with the aim of studying exposure-disease associations not easily addressed by single cohorts, a second workshop was convened by the International Agency for Research on Cancer (IARC) and the US-NCI in October 2010 in France. At this meeting the representatives from nine countries and 18 studies agreed to form an active consortium coordinated by IARC, named AGRICOH.

## 3. Why a Consortium of Agricultural Cohorts?

The purpose of AGRICOH is to promote and sustain collaboration and data sharing/pooling to assess the association between various agricultural exposures and a wide range of health outcomes with a particular focus on those associations that cannot easily be investigated in individual studies because of rare exposures (e.g., use of infrequently applied chemicals) or relatively rare outcomes (e.g., cancer, neurologic and auto-immune diseases). AGRICOH will seek to identify potential health hazards as well as protective factors that may affect both agricultural and non-agricultural populations. These aims will be supported by assembling background information from participating cohorts, developing a plan for harmonization of core exposure and outcome variables, and pooling of data, effectively increasing sample size to yield statistically powered and robust data analyses. In addition, availability of biological specimens in 16 cohorts in the consortium offers the opportunity to pool biological material in support of specific research projects with a molecular focus. Future aims of AGRICOH are to identify additional existent cohort studies focusing on agricultural populations to join the consortium and to encourage establishment of new agricultural cohorts, in low- and medium-income countries, where the range is wider and the intensity of exposures is expected to be higher, but their role in health and disease is rarely documented.

The consortium focuses on cohort studies with a broad definition of agricultural exposures and populations. These include crop and animal farming activities and environments, active and retired agricultural workers, farm owners and their families, including the occupational and the residential milieu, professional groups exposed to specific agents used in farming, such as pesticides, or generated during farming, such as organic dust produced in settings such as grain or poultry production. Availability of biological specimens is not a pre-requisite to join the consortium. The majority of cohorts in AGRICOH research health outcomes in relation to occupational and environmental exposures with a focus on agricultural settings. The Korean Multi-Center Cancer Cohort, the Janus Serum Bank of Norway and the Ontario Health Study on the other hand, are general population cohorts with the first two studies encompassing a significant number of individuals from agricultural populations and the latter having potential to oversample in agricultural areas.

## 4. Cohorts

As of February 2011, AGRICOH comprises 22 cohorts from five continents. The studies are from South Africa (1), Canada (3), Costa Rica (2), USA (6), Republic of Korea (1), New Zealand (2), Denmark (1), France (3) and Norway (3) (see Table 1). In total, ten cohorts offer data on cancer incidence: the New Grain Worker’s Study (Saskatchewan, Canada), Cancer and Mortality among Workers of Banana Plantations (Costa Rica), the Agricultural Health Study (USA), the Next Generation Cohort of Agricultural Health Study (USA), the Marshfield Epidemiologic Study Area (MESA) Farm Cohort (USA), the Korean Multi-center Cancer Cohort, the Agriculture and Cancer Cohort (France), the Janus Serum Bank (Norway), the Cancer in the Norwegian Agricultural Population Cohort and the Ontario Health Study.

Other health outcomes studied in the past or planned for future research by cohorts in AGRICOH include respiratory (15 cohorts), neurologic (nine cohorts), auto-immune (five cohorts) and cardiovascular (five cohorts) diseases as well as reproductive outcomes (seven cohorts), allergic disorders (12 cohorts) and injuries (10 cohorts) (Table 1). The cohorts contain a wide range of population sizes (from a few hundred to over half-million persons), sub-groups (men/women, adults/children/infants, rural/urban, emerging farmers) and degrees of implementation (from completed follow-up to recently initiated recruitment). Sixteen cohorts have access to biological specimens on at least a sub-sample of participants. The type and extent of characterization of agricultural exposures varies widely across these studies. The majority use questionnaires (including exposure journals) to gather most of the exposure data. A few cohorts obtain information on occupation, duration of employment and information on lifestyle factors from direct record linkage to agricultural census data and/or national public health administration databases. Personal, household, and farm environment sampling offer additional exposure data in several cohorts. A number of cohorts have the capacity to conduct exposure assessment using stored biologic specimens or through field studies. Exposure to pesticides, fertilizers, endotoxins, mycotoxins, viruses, organic and inorganic dust, diesel and other exhaust gasses, and solvents are the main exposures documented in the cohorts in AGRICOH. Published articles providing a description of the cohorts in AGRICOH, when available, are cited in Table 1.

## 5. Structure

A Steering Committee of nine members encompassing a broad spectrum of expertise, in terms of health outcomes, (agricultural) exposures assessment and data pooling experience, will lead the discussion on issues related to AGRICOH during and in between annual meetings and will guide the development of future AGRICOH activities. A coordinator from IARC (Scientific Secretariat) will provide support to the Steering Committee and consortium. Meetings dedicated to the consortium to consolidate objectives and future plans are planned to be held on an annual basis.

## 6. Future Plans

The Consortium welcomes project proposals involving pooling of data from all interested researchers. The ultimate decision about whether to contribute data and participate in any particular project rests with the individual AGRICOH consortium members, *i.e.*, the principal investigators of the cohorts. To facilitate future projects the need for data harmonization of selected core variables was discussed at the 2010 Workshop and will be initiated in May 2011. Each approved project will require some data harmonization. Eighteen research concepts for data pooling were discussed during the AGRICOH Workshop at IARC in October 2010 involving the study of cancer, respiratory, neurologic and other health outcomes in association with pesticides, organic dust and other exposures. These were supported by the consortium members and more developed proposal plans to guide data pooling in support of each of those concepts are in preparation.

AGRICOH, including 22 cohorts from nine countries in five continents welcomes new cohorts and proposals to research the association between agricultural exposures and health outcomes. Additional information on these procedures or on the consortium is featured in the webpage (http://agricoh.iarc.fr). The next annual meeting of the consortium is being planned for 12–13 September 2011 in Barcelona.

## Figures and Tables

**Table 1.** Description of cohorts in AGRICOH.

**COHORTS, COUNTRY (alphabetically by continent and country within continent)**	**ENROLLMENT, FOLLOW-UP, EXPOSURE ASSESSMENT**	Mortality	Cancer Incidence	Respiratory Diseases	Neurologic Diseases	Reproductive Outcome	Allergic Disorders	Injuries	Autoimmune Diseases	CVD	POPULATION					Biological Specimens
											Adults	Men	Women	Children	Targeted	
Pesticide exposure in emerging farmers, South Africa [a]^	2008–2009; job/residential history, quarterly pesticide exposure journals, biomonitoring										270	180	90			Blood (plasma, RBC), urine
Ontario Health Study*, Canada [b]	2009–2013; annual record linkage; occupational history; potential for detailed exposure, bio-monitoring in subset [11]										8,200*				≥150,000	Blood and urine
New Grain Workers’ Study, Canada [c]	1980–1981; every 2 yrs until 1985 [12]											335				
Grain dust medical surveillance programme, Canada [c]	1978–1993; every 3 yrs; number of years in the grain industry; practice of dust control in elevators [13]											20,831				Buccal smear
Cancer in workers in banana plantations in Costa Rica [d]	Banana plantation employment in 1972–1979; follow-up 1981–1992; duration employment [14]										34,457	29,565	4,892			
Infants and Environmental Health, Costa Rica [e]	2010–2011; baseline at pregnancy, follow-ups: 12 and 24 months. Question-naire, bio-monitoring												350	350	450	Blood; urine; hair; milk. Child: blood; urine
Farmers Health Study, USA [f]	1993–2004; 2 follow-ups: 1998, 2004. Questionnaires; dust level validation [8]										1,947	1,751	196			
MICASA Study, USA [f]	2006–2007; follow-ups: 2008–2009, 2009–2010, 2011–2012, occupational history, questionnaire										843	422	421			
Next Generation Cohort of Agricultural Health Study, USA [g]	1975–1999; parental exposure as reported via questionnaire [15]											18,263	17,114	35,414	To enroll birth years 2000–2009	Buccal cells in 45% of parents
KEOKUK County Rural Health, USA [h]	1994–2011; 2 follow-ups, job histories, occupational surveys, questionnaire, environment sampling [16]										3,002	1,426	1,576			In round 3: Buccal cells; blood; saliva
AHS private and commercial applicators, USA [i]	1993–1997; 2 follow-ups, questionnaires, including exposure to 50 pesticides; environment sampling [17]										52,394 private, 4,916 commercial applicators	55,748 applicators and 219 spouses	32,127 spouses and 1,562 applicators			Buccal cells on 40%
The Marshfield Epidemiologic Study Area (MESA) Farm Cohort, USA [j]	1991–2010; passive follow-up; farming exposures actively collected on a per project basis [4,18]										5,487	2,891	2,596			Banked DNA, serum, plasma in some adults
Korean Multi-center Cancer Cohort*, Republic of Korea [k]	1993–2004; 1 follow-up; exposure to pesticides through questionnaires[19]										19,688*	7,916	11,772			Plasma, serum, buffy, RBC, urine
Environmental exposures and asthma risk in babies born in farms, New Zealand [l]	2007–2012; 3 follow-ups, exposures and job history by questionnaire, dust indoor sampling												∼700	∼700	800	Blood (serum) from child
Asthma and atopyin farmer's children and their parents, New Zealand [l]	2001–2003; exposures, job history by questionnaire, dust and water sampling [3,20]										5,616			1,899		
SUS Study, Denmark [m]	1992–1994; 5 follow-ups, exposures by questionnaire; dust and LPS sampling [21]										2,371 407 conscripts	1,734	230			Blood (serum, buffy)
FERMA, France [n]	2006 and 2008; no follow-up completed to date; questionnaire, air sampling, bio-monitoring [22]										504			300	2000	Blood, saliva, urine
AGRICAN, France [o]	2005–2007; first follow-up in 2012–2014; exposures by questionnaire										187,471	103,135	84,336			Blood (serum, buffy, RBC), urine in 750
PHYTONER, France [p]	1997–1998; 2 follow-ups, first during 2001–2003. Job calendars, questionnaire at enrollment[23]										918	739	179			
The Janus Serum Bank*, Norway [q]	1973–2004; continuous follow-up for cancer. Occupation from census data; smoking from Nat. Inst. Public Health linkage[24]										316,951*	165,390	151,561			Blood (serum)
Cancer in the Norwegian agricultural population, Norway [r]	1969–1989; data from agricultural census; farm production records, meteo and fungal forecasts [10,25]											137,000	111,000	323,000		Members can be nested in Janus
Norway Farmer Cohort [s]	1990–1992; farms visited in 1992–1996; farming tasks and other from questionnaires; personal dust samples [26]										8,482	5,564	2,918			Blood (serum)

^ Investigators and affiliation

* General population cohort encompassing a significant number of agricultural populations or having potential to oversample agricultural areas

CVD: Cardiovascular diseases

LPS: Lipopolysaccharides

RBC: Red blood cells

[a] Leslie London, Mohamed Aqiel Dalvie and colleagues, University of Cape Town, South Africa

[b] John McLaughlin, Lyle Palmer, Paul Demers and Ontario Institute for Cancer Research, Cancer Care Ontario, Canada

[c] Punam Pahwa and colleagues, University of Saskatchewan, Canada

[d] Catharina Wesseling, Universidad Nacional, Heredia, Costa Rica

[e] Berna van Wendel, Catharina Wesseling and colleagues, Universidad Nacional, Heredia, Costa Rica

[f] Marc B. Schenker University of California at Davis, USA

[g] Chuck Lynch, Paul Romitti, Michael Alavanja, Jane Hoppin, University of Iowa, U.S. National Cancer Institute, U.S. National Institute of Environmental Health Sciences, USA

[h] James Merchant, University of Iowa, USA

[i] Michael Alavanja, Laura Beane Freeman, Dale Sandler, Jane Hoppin, U.S. National Cancer Institute, U.S. National Institute of Environmental Health Sciences, USA

[j] Stephen Waring and colleagues at Marshfield Clinic Research Foundation, USA

[k] Keun-Young Yoo, Hai-Rim Shin and colleagues at Seoul National University, Republic of Korea

[l] Jeroen Douwes and colleagues, Massey University, New Zealand

[m] Torben Sigsgaard and colleagues at Aarhus University, Denmark

[n] Isabella Annesi-Maesano and Denis Caillaud, INSERM-Paris, CHU de Clermont-Ferrand, France

[o] Pierre Lebailly and Isabelle Baldi, Centre F. Baclesse in Caen, University of Bordeaux, France

[p] Isabelle Baldi, University of Bordeaux, France

[q] Hilde Langseth and Kristina Kjærheim, Cancer Registry of Norway, Norway

[r] Karl-Christian Nordby and Petter Kristensen National Institute of Occupational Health, Norway

[s] Helge Kjuus and Wijnand Eduard, National Institute of Occupational Health, Norway
